# Clinical and Epidemiological Characteristics of 1283 Pediatric Patients with Coronavirus Disease 2019 during the First and Second Waves of the Pandemic—Results of the Pediatric Part of a Multicenter Polish Register SARSTer

**DOI:** 10.3390/jcm10215098

**Published:** 2021-10-30

**Authors:** Maria Pokorska-Śpiewak, Ewa Talarek, Anna Mania, Małgorzata Pawłowska, Jolanta Popielska, Konrad Zawadka, Magdalena Figlerowicz, Katarzyna Mazur-Melewska, Kamil Faltin, Przemysław Ciechanowski, Joanna Łasecka-Zadrożna, Józef Rudnicki, Barbara Hasiec, Martyna Stani, Paulina Frańczak-Chmura, Izabela Zaleska, Leszek Szenborn, Kacper Toczyłowski, Artur Sulik, Barbara Szczepańska, Ilona Pałyga-Bysiecka, Izabela Kucharek, Adam Sybilski, Małgorzata Sobolewska-Pilarczyk, Urszula Dryja, Ewa Majda-Stanisławska, Sławomira Niedźwiecka, Ernest Kuchar, Bolesław Kalicki, Anna Gorczyca, Magdalena Marczyńska

**Affiliations:** 1 Department of Children’s Infectious Diseases, Regional Hospital of Infectious Diseases in Warsaw, Medical University of Warsaw, 01-201 Warsaw, Poland; mpspiewak@gmail.com (M.P.-Ś.); jolanta.popielska@wum.edu.pl (J.P.); konrad.zawadka@wum.edu.pl (K.Z.); magdalena.marczynska@wum.edu.pl (M.M.); 2 Department of Infectious Diseases and Child Neurology, Poznan University of Medical Sciences, 60-572 Poznan, Poland; amania@ump.edu.pl (A.M.); mfiglerowicz@gmail.com (M.F.); katarzynamelewska@ump.edu.pl (K.M.-M.); faltinkamil@interia.pl (K.F.); 3 Department of Infectious Diseases and Hepatology, Faculty of Medicine, Collegium Medicum, Nicolaus Copernicus University, 85-030 Bydgoszcz, Poland; mpawlowska@cm.umk.pl (M.P.); m.pilarczyk@wsoz.pl (M.S.-P.); 4 Department of Paediatrics and Infectious Diseases, Regional Hospital in Szczecin, 71-455 Szczecin, Poland; przciechanowski@spwsz.szczecin.pl (P.C.); zadrozna@spwsz.szczecin.pl (J.Ł.-Z.); rudnicki@spwsz.szczecin.pl (J.R.); 5 Department of Children’s Infectious Diseases, Provincial Jan Boży Hospital in Lublin, 20-089 Lublin, Poland; bhasiec@wp.pl (B.H.); martyna.stani@janbozy.pl (M.S.); paulina.franczak@janbozy.pl (P.F.-C.); 6 Department of Paediatrics and Infectious Diseases, Wroclaw Medical University, 50-368 Wroclaw, Poland; izabela.zaleska@gmail.com (I.Z.); leszek.szenborn@umed.wroclaw.pl (L.S.); 7 Department of Pediatric Infectious Diseases, Medical University of Bialystok, 15-274 Bialystok, Poland; kacper.toczylowski@umb.edu.pl (K.T.); artur.sulik@umb.edu.pl (A.S.); 8 1st Department of Pediatrics, Collegium Medicum Jan Kochanowski University, 25-317 Kielce, Poland; g.b.szczepanski@onet.eu (B.S.); bysiecka@gmail.com (I.P.-B.); 9 2nd Department of Paediatrics, Centre of Postgraduate Medical Education, Department of Paediatrics and Neonatology with Allergology Center, Central Clinical Hospital of the Ministry of the Interior, 02-507 Warsaw, Poland; iza.orlinska@gmail.com (I.K.); adam.sybilski@cskmswia.pl (A.S.); 10 Department of Paediatric Infectious Diseases, Medical University of Lodz, 91-347 Lodz, Poland; urszuladryja@gmail.com (U.D.); emajda@lodz.home.pl (E.M.-S.); 11 Department of Paediatric Infectious Diseases, Pomeranian Center of Infectious Diseases and Tuberculosis in Gdansk, 80-214 Gdansk, Poland; sniedzwiecka@szpitalepomorskie.eu; 12 Department of Paediatrics with Clinical Assessment Unit, Medical University of Warsaw, 02-091 Warsaw, Poland; ekuchar@wum.edu.pl; 13 Department of Paediatrics, Paediatric Nephrology and Allergology, Military Institute of Medicine, 04-349 Warsaw, Poland; kalicki@wim.mil.pl; 14 The Ward of Pediatric Infectious Diseases and Hepatology, The John Paul II Hospital in Krakow, 31-202 Krakow, Poland; agorczyc@szpitaljp2.krakow.pl

**Keywords:** children, clinical presentation, coronavirus disease 2019 (COVID-19), epidemiology, severe acute respiratory syndrome coronavirus 2 (SARS-CoV-2)

## Abstract

This prospective multicenter cohort study aimed to analyze the epidemiological and clinical characteristics of coronavirus disease 2019 (COVID-19) in children. The study, based on the pediatric part of the Polish SARSTer register, included 1283 children (0 to 18 years) who were diagnosed with COVID-19 between 1 March 2020 and 31 December 2020. Household contact was reported in 56% of cases, more frequently in younger children. Fever was the most common symptom (46%). The youngest children (0–5 years) more frequently presented with fever, rhinitis and diarrhea. Teenagers more often complained of headache, sore throat, anosmia/ageusia and weakness. One fifth of patients were reported to be asymptomatic. Pneumonia was diagnosed in 12% of patients, more frequently in younger children. During the second wave patients were younger than during the first wave (median age 53 vs. 102 months, *p* < 0.0001) and required longer hospitalization (*p* < 0.0001). Significantly fewer asymptomatic patients were noted and pneumonia as well as gastrointestinal symptoms were more common. The epidemiological characteristics of pediatric patients and the clinical presentation of COVID-19 are age-related. Younger children were more frequently infected by close relatives, more often suffered from pneumonia and gastrointestinal symptoms and required hospitalization. Clinical courses differed significantly during the first two waves of the pandemic.

## 1. Introduction

Due to the rapid spread and enormous burden of coronavirus disease 2019 (COVID-19), the World Health Organization declared it a pandemic in March 2020 [1]. By 31 December 2020, almost 1,295,000 cases of COVID-19 had been diagnosed in Poland. The proportion of pediatric patients remains unknown. However, according to available data from the first two months of the pandemic, children constituted 6.68% of cases (*n* = 1191) in Poland, with an infection rate of 15.49/100,000 children, which increased with age (10.79/100,000 in children below 4 years of age to 21.59/100,000 in patients between 15 and 19 years old) [2]. From the beginning of the pandemic, available observations suggested that pediatric populations are less affected than adults, with a lower incidence and milder clinical course of the disease [3,4,5,6]. Many reports, including both observational studies [7,8,9,10,11,12] and systematic reviews [13,14,15,16], address the epidemiological and clinical characteristics of pediatric patients with COVID-19, most of which were published in the first months of the pandemic, providing valuable information about the novel disease in children. As the pandemic continues, the number of pediatric cases grows, and answering some critical questions should become easier, e.g., which children are more vulnerable to severe acute respiratory syndrome coronavirus 2 (SARS-CoV-2) infection, whether any factors can predict a more severe clinical course and whether the disease remained the same and had the same clinical picture during subsequent waves of the pandemic.

This study aimed to analyze the clinical and epidemiological characteristics of COVID-19 in children. In particular, we investigated differences in disease course according to patient age and wave of the pandemic (the first vs. the second). In addition, predictors of COVID-19-related pneumonia and gastrointestinal symptoms were analyzed.

## 2. Material and Methods

### 2.1. Study Design and Setting

This multicenter prospective cohort study based on the pediatric part of the SARSTer register (SARSTer-PED) included children (0 to 18 years) who were diagnosed with COVID-19 between 1 March 2020, and 31 December 2020. Fourteen Polish inpatient centers dedicated to pediatric patients with COVID-19 reported their cases using an electronic questionnaire addressing epidemiological and clinical data. Any patient younger than 18 years with confirmed SARS-CoV-2 infection was eligible for inclusion.

### 2.2. SARS-CoV-2 Testing

COVID-19 was diagnosed by a positive real-time polymerase chain reaction (RT-PCR) on a nasopharyngeal swab performed in certified molecular diagnostics laboratories using certified RT-PCR testing methods for SARS-CoV-2 infection. After validation and approval of second-generation antigen testing for SARS-CoV-2 infection as a reliable method for the diagnosis of COVID-19 (30 October 2020), cases confirmed by this method were also included. 

### 2.3. Data Collection and Study Definitions

Demographic data included age and sex. Epidemiologic data included known exposure to a person with confirmed SARS-CoV-2 infection (in the household or otherwise), history of international travel in the 14 days before disease onset, the duration of symptoms before presentation and any comorbidity, including bronchial asthma, cardiovascular disease, immunodeficiency, obesity, diabetes and arterial hypertension. Immunodeficiency was defined as congenital or acquired immunodeficiency or as the concurrent use of an immunosuppressive agent. Obesity was defined as body mass index ≥the 95th percentile for age/sex. All symptoms at the time of admission and during hospitalization (if applicable) were documented. Fever was defined as a body surface temperature ≥38.5 °C. Laboratory testing and imaging results (if performed due to clinical indications) were recorded. Diagnosis of pneumonia was based on clinical signs, auscultation findings and/or chest X-ray abnormalities. Criteria for hospitalization varied across the study period. In the first several weeks, due to limited availability of PCR testing and scarce experience with pediatric COVID-19, most patients were admitted to the hospital for confirmation of SARS-CoV-2 infection and clinical assessment. Later, indications for hospital admission were clinical. For the purpose of this study, two waves of the pandemic were defined: the first wave lasted from March to August 2020 and the second wave lasted from September to December 2020, reflecting the two waves of the pandemic observed in Poland.

### 2.4. Statistical Analysis 

Statistical analysis was performed using MedCalc Statistical Software version 19.2.1 (MedCalc, Ostend, Belgium, https://www.medcalc.org, accessed on 7 October 2021). Categorical variables were compared using the chi-square test. Continuous variables are presented as medians with interquartile ranges (IQRs) and were compared using the Mann–Whitney U test. A two-sided *p* value < 0.05 was considered significant. In addition, logistic regression analysis was performed. Parameters with a significant difference were included in the univariate analysis, and parameters significant in the univariate analysis were included in the multivariate analysis. The results were presented as odds ratios (ORs) and 95% confidence intervals (95% CIs). Results with a CI not including 1.0 were considered significant. 

### 2.5. Ethical Statement

The study was performed in accordance with the ethical standards in the 1964 Declaration of Helsinki and its later amendments. The local ethics committee of the Regional Medical Chamber in Warsaw approved this study (No KB/1270/20; date of approval: 3 April 2020).

## 3. Results

### 3.1. Study Group

**Figure 1:**
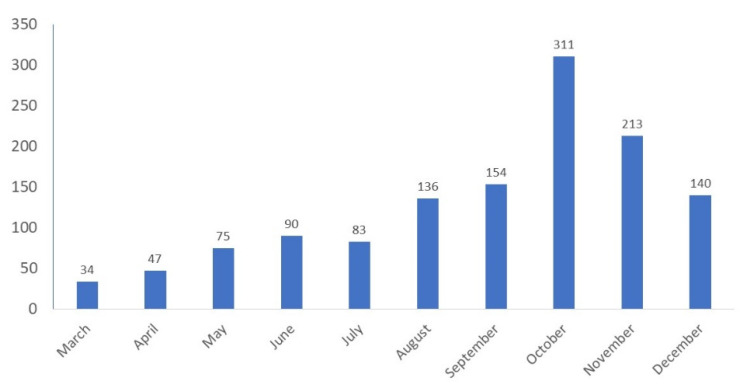
The number of COVID-19 cases in pediatric patients reported in the SARSTer-PED register throughout 2020.

Between 1 March 2020 and 31 December 2020, 1283 patients with COVID-19 were reported: 465 during the first wave of the pandemic (March to August) and 818 during the second wave (September to December, Figure 1), including 650 boys and 633 girls aged 5 days to 18 years, with a median age of 6 (1; 13) years. Among the 1283 patients, 1008 (78%) were hospitalized: 349 (35%) patients were hospitalized for no longer than 24 h and the remaining 659 (65%) patients were hospitalized for longer than 24 h, with a median hospital stay of 5 (3; 8) days. Twenty-five (2%) of the patients required oxygen therapy but none of them needed mechanical ventilation. The median duration of clinical symptoms before admission was 2 (1; 4) days. Two hundred fifty-one patients (20%) suffered from chronic comorbidities, including those potentially related to a high risk of severe COVID-19, e.g., bronchial asthma (26 patients), cardiovascular disease (22), immunodeficiency (19), obesity (8), diabetes (5) and arterial hypertension (4). Comorbidities were significantly more frequently reported in teenagers (26%) than in younger children (*p* < 0.0001) (Table 1). Three patients required admission to the intensive care unit (ICU). No fatal outcomes were reported.

Epidemiological features in the three age groups (0–5; >5–10; and >10–18 years) are presented in Table 1. Household contact with a relative infected with SARS-CoV-2 was reported in 716 (56%) cases, more frequently in younger children. Among 606 patients for whom COVID-19 was confirmed in a family member, in 260 (43%) cases, the diagnosis had been established in relatives before the children; in 281 (46%) cases, the diagnoses were established simultaneously; and in 65 (11%) cases, relatives were diagnosed after the children. Exposure other than household contact was confirmed in 83 (6%) patients, significantly more frequently in teenagers (12%), *p* < 0.0001. Forty-three (3%) patients had a history of international travel 14 days before disease onset. 

### 3.2. Clinical Presentation

**Figure 2:**
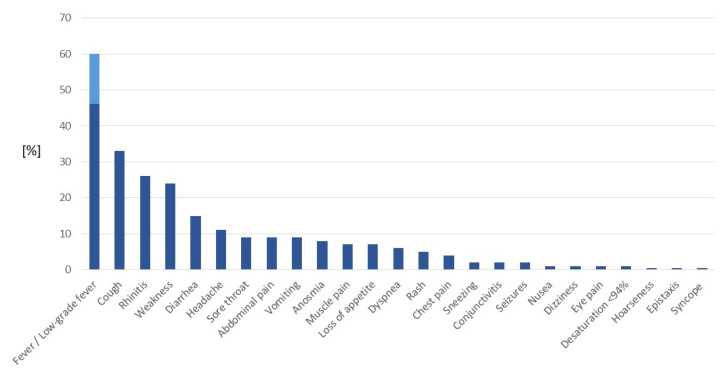
Clinical symptoms of COVID-19 in 1283 children. Data are presented as the frequency of the symptoms (%).

Fever was the most frequent symptom of COVID-19, with a prevalence of 46%. In an additional 14% of patients, low-grade fever (up to 38.5°C) was observed. The frequencies of clinical symptoms in the study group are presented in Table 1 and Figure 2. Anosmia and/or ageusia, the most specific symptoms of COVID-19, were reported in 8% of patients, including 20% of teenagers, but not in any children up to 5 years old. The youngest children (0–5 years old) more frequently presented with fever, rhinitis, diarrhea, loss of appetite and rash than teenagers, who more often complained of weakness, headache, sore throat, anosmia, muscle pain and chest pain (Table 1). In addition, dyspnea was significantly more frequently observed in teenagers (9%) than in younger children (4% in children aged 0–5 years and 3% in children aged 5–10 years, *p* = 0.0002). Two hundred seventy-one patients (21%) were reported to be asymptomatic (they were admitted for epidemiological reasons, mainly during the first weeks of the pandemic).

### 3.3. COVID-19-Related Pneumonia

Based on the clinical presentation and chest X-ray (CXR) results, pneumonia associated with COVID-19 was diagnosed in 156 (12%) patients. Typical CXR findings included bilateral patchy consolidation and ground-glass opacities with a peripheral and lower lung predominance, which were often bilateral. In our study group, children with pneumonia were younger: their median age was 18.0 (6.0; 84.5) months compared to 86.0 (20.0; 160.0) months in children without pneumonia (*p* < 0.0001). They also more frequently presented with fever, cough and gastrointestinal symptoms (Table 2). On laboratory testing, children with pneumonia had higher leukocyte counts and C-reactive protein (CRP), D-dimer, interleukin-6 (Il-6) and alanine as well as aspartate aminotransferase (ALT and AST) levels than patients without pneumonia. Interestingly, the presence of comorbidities did not cause a higher risk of COVID-19-related pneumonia (Table 2).

### 3.4. Gastrointestinal (GI) Symptoms

A total of 295 (23%) patients presented with at least one GI symptom (abdominal pain, diarrhea or vomiting). These children were younger than the remaining group without GI presentation (median age 36 vs. 87 months, *p* < 0.0001), and presented more frequently with fever and higher CRP, D-dimer and AST levels. No difference in the GI symptom incidence was noted between patients with comorbidities and otherwise healthy children (Table 3).

### 3.5. Comparison of the Clinical Presentation of COVID-19 between the First and Second Pandemic Waves

We found significant differences in the course of COVID-19 and epidemiological features among patients reported during the second wave of the pandemic compared to the patients from the first wave (Table 4). Children diagnosed during the second wave were younger, with a median age of 53 vs. 102 months (*p* < 0.0001) and required significantly longer hospitalization (*p* < 0.0001). Household exposure to SARS-CoV-2 was reported less frequently (*p* < 0.0001), whereas another source of exposure was confirmed more often. Significantly fewer asymptomatic patients were noted, whereas COVID-19-related pneumonia, GI symptoms and most other clinical symptoms were more common (Table 4).

## 4. Discussion

This study presents our experience with COVID-19 in 1283 patients from 14 pediatric hospital settings in Poland. Children aged 0–5 years accounted for a high proportion of our patients (45%), similarly to preliminary data from Italy [9], but other authors reported a higher prevalence among older children [8,11,16]. Both sexes were equally affected, whereas in a number of studies a slight male predominance has been reported [5,7,10,11,14,17]. Almost 80% of patients were hospitalized. This proportion was significantly higher during the second wave than during the first wave (84% vs. 69%, respectively, *p* < 0.0001). The difference might be caused by the fact that testing for SARS-CoV-2 during the first weeks/months of the pandemic was available in Poland mainly in hospital settings; thus, some children with suspected SARS-CoV-2 infection were referred to the hospital for confirmation of the infection. Later, access to testing was easy in primary care; thus, children with confirmed SARS-CoV-2 infection were referred to the hospital for clinical indications, which may also explain the higher proportion of asymptomatic patients during the first pandemic wave than during the second wave (36% vs. 13%, respectively, *p* < 0.0001). Young children (5 days–5 years old) were also admitted to the hospital more frequently than those older than 5 years. The possible explanation may be a higher tendency for parents to seek medical care for younger children and/or a higher tendency for clinicians to admit them to the hospital. Younger age is recognized as a risk factor of a more severe course of some infections, e.g., influenza. In this age group of our cohort pneumonia and gastrointestinal symptoms were significantly more frequent, which may suggest need of supportive treatment, even if it does mean a severe clinical course. Hospitalization rates were reported only in a limited number of previous studies, which varied from 11% to 74% during the first few months of the pandemic [4,5,7,10,11,18]. Most hospitalizations were short, which is consistent with other observations [7,10], although some systematic reviews reported a mean LoHS exceeding 10 days [14,16]. The need for a stay longer than 7 days was reported in 17% of our inpatients; these children were significantly younger (median age 52 vs. 78.5 months, respectively, *p* = 0.001) and more commonly had comorbidities (38% vs. 18%, respectively, *p* < 0.0001). According to other studies, 1 to 18% of pediatric patients required admission to the intensive care unit (ICU) [4,11,14,17]. In our cohort, only three children (< 1%) were hospitalized in the ICU.

Most children were infected in the household, and exposure to a family member with SARS-CoV-2 infection, mainly a parent, was confirmed in 52–76% of pediatric patients [3,9,10,11,14]. Our results support these observations for the first pandemic wave, when 78% of patients had a history of household exposure compared with 46% during the second wave (*p* < 0.0001). Analysis of age groups revealed that household exposure was less common in children >10–18 years old, which may be explained by the greater mobility of teenagers, even in lockdown conditions when schools were closed. Although predominant community exposure (60%) was reported in South America by Antunez-Montes et al., they explained that this result was due to the lack of a strict lockdown [19].

The pediatric population is not commonly affected by comorbidities compared to adults, but children with chronic underlying conditions were reported in all studies. At least one comorbidity was reported in 20 to 45% of pediatric patients with COVID-19 [4,7,9,11,14]. The most common condition was chronic pulmonary disease, including bronchial asthma. In the present cohort, the prevalence of comorbidities was 20%, with bronchial asthma as the most commonly reported condition. Comorbidities were significantly more widely reported among patients during the second wave (24% vs. 15%, respectively, *p* < 0.0001). Some authors found that comorbidities (e.g., pulmonary, endocrine, neurologic and immunocompromising conditions) increase both the odds of hospitalization and the need for respiratory support [7,11], but these observations are not confirmed by others [10] or our results. It seems that comorbidity in a patient with SARS-CoV-2 infection may be a cause for referral to the hospital rather than a cause of a more severe clinical course of the disease, since COVID-19-related pneumonia was not more frequent than among patients with no underlying conditions.

An asymptomatic course of SARS-CoV-2 infection was observed in 2.5–19% of patients [7,9,11,12,14,16]; in this study, an asymptomatic course was found in 21% of the whole cohort, with a significant difference between the first and second waves of the pandemic (36% vs. 13%, respectively, *p* < 0.0001). Different diagnostic approaches may explain this result during the first months of the pandemic when testing for SARS-CoV-2 infection was partially performed for epidemiologic purposes regardless of the clinical presentation. In the second wave, as the number of COVID-19 cases significantly increased, patients were tested mainly because of clinical suspicion of the disease. The proportion of asymptomatic cases was age-related, with approximately one third in children > 5–10 years old. Among symptomatic patients, the most commonly reported signs were fever (46%), followed by a cough (33%), rhinitis (26%) and weakness (24%); these same symptoms were the most common in a systemic review including 7780 pediatric patients conducted by Hoang et al., although their proportions varied (59%, 56%, 20% and 19%, respectively) [14]. When comparing the prevalence of these symptoms in different age groups, we found that fever and rhinitis were significantly more common in children aged 0–5 years and weakness in those aged > 10–18 years. The relationship between age and the prevalence of symptoms was demonstrated in a UK pediatric study [17] and a multinational study with both pediatric and adult populations [20]. In contrast to others, Badal et al. reported headache as the most common symptom, which was present in 60–74% of pediatric patients [16]. In our whole cohort and in the >10–18 years group the prevalence rates of headache were 11% and 22%, respectively. GI symptoms, e.g., the presence of at least one of the following: vomiting, diarrhea or abdominal pain, were observed in 23% of patients, which is similar to data reported by others (22–24%) [9,11]. GI symptoms had the highest prevalence (30%) in children aged 5 days–5 years. Patients with GI symptoms were significantly younger (36 vs. 87 months, respectively, *p* < 0.0001) and more commonly presented with fever and higher levels of inflammatory markers: leukocyte count, CRP and D-dimer. Comorbidities were not a predictor of a GI presentation. When comparing the two pandemic waves, GI symptoms were approximately 2.5 times more frequent during the second wave. At that time, Polish pediatricians (and parents) were more aware of both COVID-19 and pediatric inflammatory multisystem syndrome temporally associated with SARS-CoV-2 (PIMS); thus, children with abdominal pain and diarrhea (especially accompanied by fever), were tested for SARS-CoV-2 infection due to the suspicion of PIMS.

Pneumonia is a relatively common clinical feature in children with COVID-19, which is reported in 25–65% of pediatric patients [21]. Due to the heterogeneity of the data, including the basis of diagnosis (clinical symptoms vs. chest imaging), comparisons are difficult. According to studies published early in the pandemic, chest imaging (mainly CXR) was also performed in asymptomatic children. Patients with mild clinical symptoms and abnormalities were found in some proportions of these studies [3,12]. As knowledge about the presentation and evolution of COVID-19-related pneumonia in pediatric patients increased, indications for chest imaging were limited [22]. In our cohort, pneumonia was diagnosed in a relatively low proportion of patients (12%). Of note, patients with comorbidities were not at a greater risk in contrast to children presenting GI symptoms during the clinical course. Pneumonia was more frequent during the second wave of the pandemic than during the first wave.

Loss of smell and/or taste, which appeared in the first months of the pandemic as a specific and common symptom of SARS-CoV-2 infection in adults [23,24], was also observed in children [18,25]. In one American cohort study, loss of smell was reported in 6% of pediatric patients, but in others, anosmia or ageusia was not listed among clinical presentations. The prevalence of these symptoms was 8% in our cohort, which reached 20% in the oldest age group (> 10–18 years old). The prevalence also seemed higher during the first vs. the second wave of the pandemic, although without statistical significance (10% vs. 7%, respectively, *p* = 0.12).

The differences in the clinical course of COVID-19 between the two waves of the pandemic suggest the probable influence of new variants of SARS-CoV-2 on disease presentation. However, as the first cases of infection caused by SARS-CoV-2 variants were reported in Poland in January and February 2021, we are not able to analyze the possible influence of different mutations on the clinical course of COVID-19 in the described period of 2020.

This study has limitations. It was conducted in hospital settings, and the spectrum of pediatric COVID-19 may have been affected by the patient population. Children referred to the hospital are more likely to exhibit more symptoms and be more ill. The prevalence of some symptoms is challenging to assess among young children since they are not objective or are difficult for a patient to describe and may be under- or overestimated by caregivers. All collaborating centers used the same questionnaire for data collection but clinical management, e.g., admission criteria, could vary. Especially during the second wave of the pandemic, access to primary medical care in Poland was limited and this might have affected decisions regarding hospitalization. The main advantages of our study include the prospective design and the inclusion of a substantial number of cases divided into age groups, which allowed us to demonstrate that the presentation of COVID-19 is age-related. In addition, to the best of our knowledge, this is the first description of differences in the clinical course of COVID-19 in children between the first and second waves of the pandemic. Since the vast majority of previous studies present data from the first months of the pandemic, our observations made for several months may extend experience with pediatric COVID-19.

In conclusion, based on our experience, the epidemiological characteristics of pediatric patients infected with SARS-CoV-2 and the clinical presentation of COVID-19 are age-related. Sources of infection seem to differ according to patient age. Younger children are more frequently infected by close relatives, and they more often suffer from pneumonia and gastrointestinal symptoms and require hospitalization, which is usually short. In addition, the clinical course of the disease differed significantly during the first two waves of the pandemic, suggesting the possible influence of new variants of SARS-CoV-2 on disease presentation.

## Figures and Tables

**Table 1:** Clinical presentation and epidemiological characteristics of COVID-19 in the study group and in different age groups.

Characteristics	Total N = 1283	Age (Years)			p *
		0–5 N = 589	>5–10 N = 225	>10 N = 469	
	*n* (%)	*n* (%)	*n* (%)	*n* (%)	
Epidemiological: Household contact with an infected family member	716 (56)	348 (59)	149 (66)	219 (47)	<0.0001
Confirmed other contact	83 (6)	11 (2)	18 (8)	54 (12)	<0.0001
History of international travel (during 14 days before disease onset)	43 (3)	9 (2)	4 (2)	30 (6)	<0.0001
Comorbidities Bronchial asthma Cardiovascular disease Immunodeficiency Obesity Diabetes Arterial hypertension	251 (20) 26 (2) 22 (1.7) 19 (1.5) 8 (0.6) 5 (0.4) 4 (0.3)	84 (14) 5 (0.8) 8 (1) 5 (0.8) 0 0 0	43 (19) 7 (3) 2 (1) 8 (4) 0 0 0	124 (26) 14 (3) 12 (3) 5 (1) 8 (2) 5 (1) 4 (1)	<0.0001 0.005
Number of patients during 1st/2nd waves of the pandemic	465 (36)/ 818 (64)	155 (26)/ 434 (74)	116 (52)/109 (48)	194 (41)/275 (59)	<0.0001
Clinical Presentation					
Fever	597 (46)	351 (60)	91 (40)	155 (33)	<0.0001
Cough	417 (33)	209 (35)	46 (20)	162 (35)	0.0001
Rhinitis	339 (26)	190 (32)	36 (16)	113 (24)	<0.0001
Weakness	305 (24)	120 (20)	53 (19)	142 (30)	0.0002
Diarrhea	191 (15)	128 (22)	20 (9)	43 (9)	<0.0001
Headache	139 (11)	6 (1)	34 (15)	101 (22)	<0.0001
Sore throat	114 (9)	19 (3)	18 (8)	129 (28)	<0.0001
Abdominal pain	118 (9)	42 (7)	36 (16)	40 (9)	<0.0001
Vomiting	111 (9)	59 (10)	23 (10)	29 (6)	0.05
Anosmia/ageusia	104 (8)	0 (0)	11 (5)	92 (20)	<0.0001
Muscle pain	96 (7)	9 (2)	12 (5)	75 (16)	<0.0001
Loss of appetite	93 (7)	78 (13)	9 (4)	6 (1)	<0.0001
Dyspnea	76 (6)	26 (4)	6 (3)	44 (9)	0.0002
Rash	70 (5)	47 (8)	9 (4)	14 (3)	0.001
Chest pain	47 (4)	1 (<1)	3 (1)	43 (9)	<0.0001
Seizures	24 (2)	13 (2)	4 (2)	5 (1)	0.36
Pneumonia related to COVID-19	156 (12)	112 (19)	13 (6)	31 (7)	<0.0001
Gastrointestinal symptoms	295 (23)	175 (30)	47 (21)	73 (16)	<0.0001
Asymptomatic course	271 (21)	97 (16)	69 (31)	105 (22)	<0.0001
Hospitalization	1008 (79)	500 (85)	165 (73)	343 (73)	<0.0001

Data are presented as a number (%); * *p* values were calculated for the three age groups (0–5 vs. > 5–10 vs. > 10 years). 3.2. Epidemiological Characteristics

**Table 2:** Clinical factors associated with COVID-19-related pneumonia.

Factor	Pneumonia		*p*	OR (95% CI)
	With Pneumonia (*n* = 156)	Without Pneumonia (*n* = 1127)		
Age (months)	18.0 (6.0; 84.5)	86.0 (20.0; 160.0)	<0.0001	0.99 (0.98–0.99)
Sex	M: 89 (57) F: 67 (43)	M: 561 (50) F: 566 (50)	0.08	-
Fever (>38.5 °C)	103 (66)	494 (44)	<0.0001	1.48 (1.17–1.88)
Cough	79 (51)	338 (30)	<0.0001	2.06 (1.47–2.88)
Gastrointestinal symptoms	46 (29)	249 (22)	0.04	1.47 (1.01–2.14)
Leukocyte count (10^3^/μL)	8.9 (6.4; 12.6)	7.4 (5.3; 10.9)	0.0005	-
CRP (mg/L)	6.0 (2.4; 27.8)	3.0 (0.7; 10.6)	<0.0001	1.01 (1.01–1.01)
Interleukin-6 (pg/mL)	8.9 (2.8; 43.6)	4.5 (2.2; 9.9)	0.02	-
D-dimer (ng/mL)	451.0 (230.0; 980.0)	311.0 (205.0; 486.2)	0.0004	-
ALT (IU/L)	22.0 (15.3; 34.7)	17.0 (13.0; 25.0)	<0.0001	-
AST (IU/L)	41.0 (30.0; 53.0)	32.0 (24.0; 42.0)	<0.0001	-
Comorbidities *	36 (23)	232 (21)	0.47	-

Data are presented as the median (IQR) or *n* (%), as appropriate. ALT—alanine aminotransferase; AST—aspartate aminotransferase; CI—confidence interval; CRP—C-reactive protein; and OR—odds ratio. * Comorbidities included: bronchial asthma, cardiovascular disease, immunodeficiency, obesity, diabetes, arterial hypertension and other.

**Table 3:** Clinical factors associated with gastrointestinal symptoms in the course of COVID-19.

Factor	Gastrointestinal (GI) Symptoms (Abdominal Pain, Diarrhea or Vomiting)		*p*	OR (95% CI)
	Patients with GI Symptoms (*n* = 295)	Patients without GI Symptoms (*n* = 988)		
Age (months)	36 (9; 120)	87 (20; 164)	<0.0001	0.99 (0.99–0.99)
Sex	M: 153 (52) F: 142 (48)	M: 497 (50) F: 491 (50)	0.63	-
Fever (> 38.5 °C)	192 (65)	405 (41)	<0.0001	2.58 (1.96–3.38)
Leukocyte count (10^3^/μL)	8.8 (6.1; 12.8)	7.3 (5.3; 10.6)	<0.0001	-
CRP (mg/L)	5.2 (1.3; 21.0)	3.0 (0.6; 9.8)	0.0002	-
Interleukin-6 (pg/mL)	4.2 (2.1; 18.5)	4.8 (2.6; 10.6)	0.97	-
D-dimer (ng/mL)	443.5 (266.3; 739.7)	290.9 (194.3; 481.8)	<0.0001	-
ALT (IU/L)	17 (13; 26)	17 (13; 26)	0.69	-
AST (IU/L)	35 (27; 46)	32 (24; 44)	0.02	-
Comorbidities	64 (21)	204 (21)	0.69	-

Data are presented as the median (IQR) or *n* (%), as appropriate. ALT—alanine aminotransferase; AST—aspartate aminotransferase; CI—confidence interval; CRP—C-reactive protein; and OR—odds ratio.

**Table 4:** Comparison of the clinical presentation of COVID-19 in children between the 1^st^ and 2^nd^ waves of the pandemic.

Clinical Factor	Patients Diagnosed between March and August 2020 N = 465	Patients Diagnosed between September and December 2020 N = 818	*p*	OR (95% CI)
Age (months) Median (IQR)	102.0 (44.0; 154.7)	53.0 (9.0; 156.0)	<0.0001	0.99 (0.99–0.99)
Sex (male/female)	223 (48)/242 (52)	427 (52)/391 (48)	0.14	-
Hospitalized	321 (69)	687 (84)	<0.0001	2.29 (1.74–3.01)
Duration of hospitalization (days), median (IQR)	1 (1; 3)	3 (1; 6)	<0.0001	1.07 (1.04–1.11)
Household contact with an infected family member	360 (78)	355 (43)	<0.0001	0.39 (0.31–0.49)
Confirmed other contact	5 (1)	43 (5)	0.0001	-
International travel during the 14 days before symptom onset	31 (7)	12 (1)	<0.0001	0.20 (0.10–0.40)
Comorbidities	70 (15)	198 (24)	0.0001	1.72 (1.27–2.31)
CLINICAL PRESENTATION				
Asymptomatic disease course	167 (36)	104 (13)	<0.0001	0.26 (0.19–0.34)
Pneumonia related to COVID-19	42 (9)	114 (14)	0.009	1.63 (1.12–2.38)
Gastrointestinal symptoms	58 (12)	237 (29)	<0.0001	2.87 (2.10–3.94)
Fever	152 (33)	445 (54)	<0.0001	2.30 (1.81–2.93)
Cough	109 (23)	308 (38)	<0.0001	1.86 (1.43–2.41)
Rhinitis	79 (17)	260 (32)	<0.0001	2.17 (1.63–2.89)
Weakness	68 (15)	237 (29)	<0.0001	2.25 (1.66–3.03)
Diarrhea	40 (9)	151 (18)	<0.0001	2.27 (1.57–3.29)
Headache	39 (8)	102 (12)	0.02	1.48 (1.01–2.19)
Sore throat	41 (9)	73 (9)	0.94	-
Anosmia	45 (10)	59 (7)	0.12	-
Vomiting	19 (4)	92 (11)	<0.0001	2.74 (1.66–4.52)
Abdominal pain	18 (4)	100 (12)	<0.0001	3.16 (1.91-5.25)
Loss of appetite	9 (2)	84 (10)	<0.0001	6.51 (3.24-13.09)
Muscle pain	26 (6)	70 (9)	0.05	-
Dyspnea	19 (4)	57 (7)	0.03	-
Rash	20 (4)	50 (6)	0.17	-
Chest pain	13 (3)	34 (4)	0.21	-
Conjunctivitis	7 (2)	18 (2)	0.38	-
Seizures	3 (1)	21 (3)	0.01	-

Data are presented as a number (%) unless otherwise indicated. CI—confidence interval and OR—odds ratio.

## Data Availability

The datasets used and analyzed during the current study are available from the corresponding author upon reasonable request.
